# Temperature Dependence of the Complexation Mechanism of Celecoxib and Hydroxyl-β-cyclodextrin in Aqueous Solution

**DOI:** 10.3390/pharmaceutics6030467

**Published:** 2014-08-13

**Authors:** Po-Chiang Chiang, Yue Shi, Yong Cui

**Affiliations:** 1 Small Molecule Pharmaceutical Sciences, Genentech, Inc., 1 DNA Way, South San Francisco, CA 94080, USA; 2 Department of Biomedical Engineering, The University of Texas at Austin, Austin, TX 78712, USA; E-Mail: shiyue8638@gmail.com; 3 Hisun–Pfizer, Shanghai 200041, China; E-Mail: Yong.Cui@hisun-pfizer.com

**Keywords:** molecular dynamics simulation, transition state, hydroxypropyl-β-cyclodextrin, celecoxib, solubilization, complexation

## Abstract

Hydroxypropyl-β-cyclodextrin (HP-β-CD) is commonly used as a complexation reagent to solubilize compounds with poor aqueous solubility to improve *in vivo* dosing. However, the degree of solubility enhancement was often limited by the formation of only a 1:1 complex and a low complexation constant (K). Such a limitation can be significantly improved by the formation of 1:2 complexes in some cases. Despite the understanding of the solubility advantage of the formation of the 1:2 complexes, there is no systematic understanding that could drive for the formation of 1:2 complexes. Thus, in most cases, the formation of 1:2 complexes was limited by observation bases. In this study, we pioneer the usages of molecular dynamics (MD) simulation to understand the phenomena of a model drug of celecoxib (CCB) and HP-β-CD. It has been reported that celecoxib (CCB) forms 1:1 complexes with cyclodextrin in solution; however, some data suggest the existence of a 1:2 complex. The simulation results suggest that a transition state of CCB and HP-β-CD may exit at a higher temperature of CCB and HP-β-CD; a model drug, such as celecoxib (CCB), that is known to form 1:1 complexes can achieve a higher degree of complexation (1:2) and obtain much improved solubility when the same amount of cyclodextrin was used and demonstrated *in vitro*. The simulation results of CCB and HP-β-CD could be a model system that may provide important insights into the inclusion mechanism.

## 1. Introduction

Time and resource constraints increasingly often necessitate early decision-making to accelerate or stop pre-clinical drug discovery programs. Early discovery drug candidates may be potent inhibitors of new targets, but all too often exhibiting poor pharmaceutical and pharmacokinetic properties that have limited *in vivo* exposure, hence often making early assessment of *in vivo* efficacy and target safety very challenging. In the pre-clinical setting, one of the biggest problems for oral drug delivery is the solubility and dissolution rate-limited absorption. When such an issue is encountered, enabling formulations are often used to help solubilize compounds. Cyclodextrin (CD) inclusion is a technique widely used in the pharmaceutical industry to enhance drug solubility [1,2,3]. Yet, the mechanistic nature and driving forces remain fragmentary for some critical characteristics of the inclusion complexes, which include the stoichiometric ratios of the complexes, the inclusion efficiency or the complex stability and the role of cyclodextrin self-aggregation in the complex formation [4,5]. Inconsistent observations in both experimental and modeling studies are not uncommon. For instance, imipramine was determined by means of conductometric studies to form an inclusion complex by a 1:1 stoichiometry with beta cyclodextrin (β-CD) in water solution [6,7]. Other experimental studies using FTIR-HATR (horizontal attenuated total reflectance) spectroscopy, NMR and dynamic light scattering, however, led to a conclusion that complexes of a molecular ratio of 1:2 and 2:1 co-existed with a 1:1 complex in the system [8]. Furthermore, a molecular docking study showed that the complex should have a 1:2 stoichiometry (drug/β-CD) [9]. Likewise, Ventura *et al.* [10] showed through ^1^H-NMR study that celecoxib (hereafter referred to as celecoxib (CCB)) formed a 1:1 inclusion complex with dimethyl-β-cyclodextrin (DM-β-CD) in aqueous solution, while the solubility phase diagram in the same study was an A_p_-type, suggesting the formation of a higher order inclusion complex. Conversely, in another paper [11], the phase solubility profile of CCB in the presence of β-CD indicated the formation of a 1:1 stoichiometric CCB/β-CD complex, while the molecular docking study in the same paper supported a 1:2 inclusion complex in aqueous solution. The widespread inconsistency in the literature reflects the complexity of the CD inclusion mechanism and, thereby, the ambiguity arising from the determination of the complexation stoichiometry [5]. Therefore, it is crucial to clarify and reconcile these seemingly contradictory observations, so that a unifying understanding of the complexation mechanism can be delivered. We initiated and pioneered a molecular dynamics (MD) simulation study to probe the structural and energetic features of the complexation process.

The study employed a model system consisting of CCB and the most widely used CD, hydroxypropyl-β-cyclodextrin (HP-β-CD), in aqueous solutions. As mentioned above, conflicting study results were reported with regard to the CCB/CD systems, which warrant further clarification. Furthermore, the phase solubility data reported earlier on the CCB/DM-β-CD system [10] suggested that the stoichiometry of the inclusion complex appeared to be temperature dependent in the range of 25–37 °C, with the stability constant of the 1:1 complex decreasing and that of the 1:2 complex increasing with temperature. This result suggests that the complexation of CCB and CD may involve a transition stage in this temperature range, which could be a model system that may provide important insights into the inclusion mechanism. The goal of this study was to combine the experimental data with simulation and to further characterize this system to provide insights into the complexation mechanism and to clarify the ambiguity around the complexation stoichiometry.

## 2. Experimental

### 2.1. Materials

**Figure 1:**
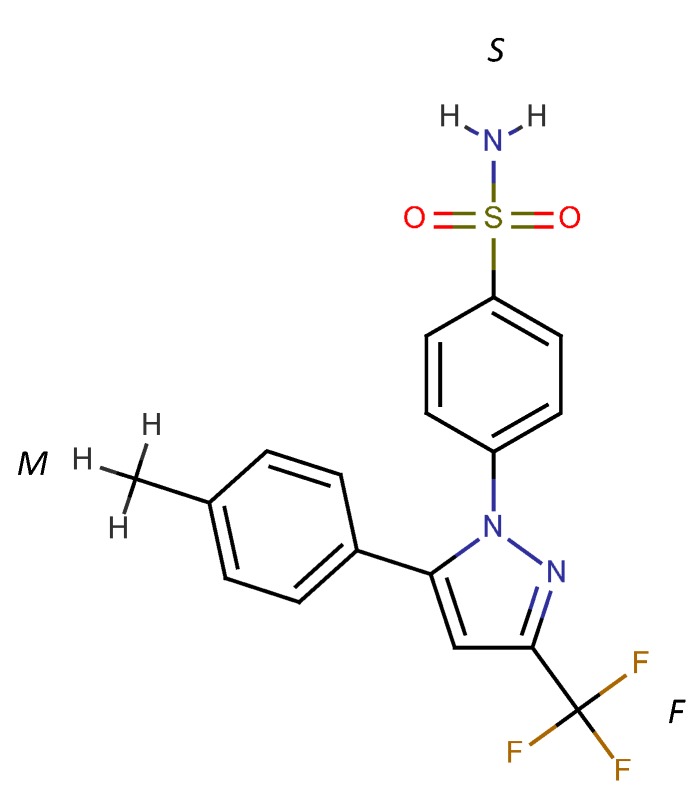
Chemical structure of celecoxib. Three ring structures are denoted as “F” for the trifluoromethyl pyrazole ring, “S” for the benzenesulfonamide ring and “M” for the methylphenyl ring.

CCB (structure presented as Figure 1) and hydroxypropyl-β-cyclodextrin (hereafter referred to as HP-β-CD, with an average molecular weight of 1,460) were obtained from Sigma–Aldrich, Co. (St. Louis, MO, USA). HPLC-grade acetonitrile was obtained from Burdick & Jackson (Muskegon, MI, USA). The water purification system used was a Millipore Milli-Q system (Millipore, MA, USA). All other chemicals used for system validation were obtained from Sigma–Aldrich and were used without further purification.

### 2.2. Phase Solubility Studies

The determination of the phase solubility profiles of CCB in the HP-β-CD solutions followed Higuchi and Connors’ method [11]. For the HP-β-CD in water solutions, the cyclodextrin and water were mixed on a weight-by weight (*w*/*w*) basis, and the density of each solution was measured by using pycnometer. Several HP-β-CD–water solutions were prepared for the study. They were 0%, 1%, 5%, 10%, 20%, 30% and 50% (0–403 mM). For the sample preparation in general, an excess amount of bulk drug was added into individual sample tubes contains 5 mL of each of the above solution and sealed and allowed to equilibrate for 24 h on a rotary shaker at room temperature (25 °C or 298 °K, hereafter, using 298 °K). After 24 h, 2.5 mL of the each resulting mixture were transferred into different sample tubes with clear labels. Extra drug was added into samples (to ensure saturation during the heating process) and sealed well with a weight check. The above mixtures were heated at 60 °C or 333 °K (hereafter, using 333 °K) for a period of 30 min with shaking occasionally and then cooling to the room temperature and checking for any weight lost (to ensure no water evaporation). The leftover mixtures and heated mixtures were then placed on a rotary shaker and equilibrated for a period of one month at room temperature, 298 °K. At the end of one month, samples were removed from the shaker and allowed to equilibrate on the bench for another week and then centrifuged at 14,000 rpm for 3 h to collect the supernatants. The supernatants were then filtered through a 0.45-μm filter and were diluted appropriately with acetonitrile prior to concentration determination by HPLC. The HPLC measurement was triplicated for each sample, and the average value was used for the solubility profiles. Solid leftovers were run on the powder X-ray diffraction (PXRD) to ensure no solid form change.

The HPLC system used was an Agilent HP 1100 HPLC (Agilent, Palo Alto, CA, USA) equipped with a diode array and variable wavelength UV detectors and a quaternary solvent delivery system (Agilent). The water purification system used was a Millipore Milli-Q system. PXRD patterns were recorded at room temperature with a Rigaku (Rigaku, The Woodlands, TX, USA) MiniFlex II Desktop X-ray Powder Diffractometer. Radiation of Cu *K*α at 30 kV–15 mA was used with a 2θ increment rate of 3°/min. The scans were run over a range of 2°–40° 2θ with a step size of 0.02° and a step time of 2 s. The powder samples were placed on a flat silicon zero background sample holder.

### 2.3. Computational Method

The highest solubility of CCB (*M*_r_ 381.4) observed in this study was approximately 0.04 M in 0.4 M HP-β-CD aqueous solution (neutral pH) for samples that were heated to 333 °K for 30 min (hereafter, referred to as 333-°K samples). The concentration of HP-β-CD in this solution was the highest (~0.4 M, or 50% g/g) in this study. This system translated to an approximate molar ratio of CCB:HP-β-CD:H_2_O = 1:10:800. With the HP-β-CD concentration reducing, the molar ratio of CCB:HP-β-CD:H_2_O of saturated CCB solutions decreased accordingly, *i.e.*, larger numbers of HP-β-CD and water molecules were needed to dissolve one CCB molecule. For instance, the solubility of CCB in 0.23 M (~30% g/mL) HP-β-CD solution (neutral pH) at 333 °K was equivalent to an approximate molar ratio of CCB:HP-β-CD:H_2_O = 1:18:3300. Based on these experimental results, while bearing in mind the limited computation resource, we constructed as our primary model a system consisting of one CCB and three HP-β-CD randomly dispersed in 3000 water molecules. With a pKa value of about 11 [10], the CCB molecule was treated as unionized in the model in accordance with the experimental condition of pH 7. The molecular structure of CCB was taken from the Cambridge Structure Database (Code: DIBBUL). For HP-β-CD, the β-cyclodextrin structure from the Cambridge Structure Database (Code: BCDEXD10) was modified by randomly substituting several hydrogen atoms of the hydroxyl groups with the hydroxypropyl groups. Based on the average molecular weight (1460) of the HP-β-CD used in this study, the degree of substitution was estimated to be approximately 5.5 per HP-β-CD molecule. We therefore rounded up to six hydroxypropyl groups per HP-β-CD molecule, with two substitutions on the primary OH (*i.e.*, OH-6) and the other four on the secondary OH (*i.e.*, OH-2 and OH-3) of the glucopyranose units. Other substitution modes, including 3/3, 0/6 and 6/0 (the substitution number on the two sides of the CD) were constructed, and no difference in the initial runs was found.

The solution model was set up using the Amorphous Cell module of the Material Studio 5.0 software package [12]. A 12-Å cubic simulation box with periodic boundary conditions in all directions was constructed with a density of 1 g/cm^3^ and a side length of about 47 Å. Two solutes, CCB and HP-β-CD, were randomly dispersed in water molecules by a modified Markov process, followed by geometric optimization of molecular configurations utilizing the COMPASS force field. Three starting structures of the solution were generated and were used for the subsequent molecular dynamics (MD) simulation. COMPASS is a Class II *ab initio* force field designed for use with organic molecules and optimized for the simulation of condensed phases (see [13] for the parameterization and validation of this force field).

The simulation was conducted in two steps. The first step was the MD simulation carried out under isothermal–isobaric (NPT) conditions in the Forcite module of Material Studio. The COMPASS force field was used to calculate both van der Waals and electrostatic interactions. The van der Walls interaction was calculated using atom-based summation with a cut-off distance of 12.5 Å, while the electrostatic interaction used Ewald summation. The time step was 1 fs, and the frame output was every 1 ps. The system pressure was held at 100 kPa by employing a Berendsen barostat. The simulation was repeated three times under two temperatures, 298 and 333 °K, respectively. The three starting structures of the solution system generated earlier were used in the MD simulation. A Nose thermostat was employed to hold the temperature constant. Structure data analysis was performed using the Forcite Analysis function.

The MD simulations resulted in several CCB/HP-β-CD complex structures, including the 1:1 and 1:2 inclusion complexes. To elucidate their relative stability, we took these structures and conducted constant velocity steered molecular dynamics (SMD) [14] simulations to probe energetic changes upon the inclusion complexation process. The initial structures were constructed based on the three representative binding modes from 1:1 inclusion and one from 1:2 inclusion complexes. For the 1:1 inclusion systems, the CCB molecule was gradually pulled out of the HP-β-CD. For the 1:2 inclusion system, the two HP-β-CD molecules were pulled away from each other first. The CCB molecule was then pulled out of the closely attached HP-β-CD. A pulling velocity of 1 Å/ns was applied to all of the SMD simulations. For each system, 20 ns simulations were performed with three repeats using the Amber 10 [15] software package. The CCB and HP-β-CD were parameterized with the AMBER GAFF force field [16] via the ANTECHAMBER module in Amber. The Three-site model (TIP3P) water model [17] was used to describe the solvent molecules. The equation of motion was integrated with a time step of 2 fs using a SHAKE algorithm. The van der Waals interactions were calculated using a cutoff distance of 12.5 Å, and the long-range electrostatic interactions were treated using the particle mesh Ewald (PME) method to replace the direct summation of interaction energies between point particles [18]. The systems were investigated using the NPT ensemble (100 kPa) at 298 and 333 °K, respectively. After SMD simulations, the potential of mean force (PMF) was reconstructed based on the Jarzynski equality [19,20]. The details of the PMF reconstruction method can be found in [21,22,23].

## 3. Results and Discussion

### 3.1. Phase Solubility

**Figure 2:**
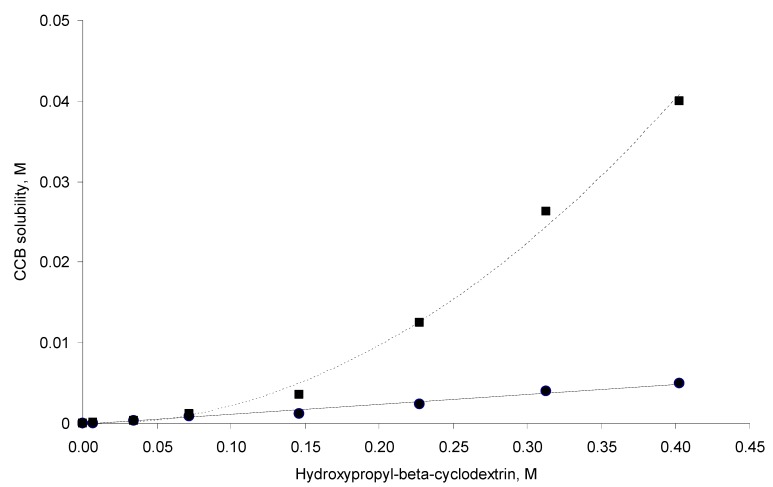
Solubility phase profiles of the celecoxib (CCB)–hydroxypropyl-β-cyclodextrin (HP-β-CD) system at 298 °K (solid circles) and 333 °K (squares).

The phase solubility profiles are shown in Figure 2. For samples that were prepared at 298 °K (hereafter, referred to as 298 °K samples), a linear curve was found (A_L_-type systems). This suggests the formation of a first-order complex between CCB and HP-β-CD. In contrast, a positive deviation from linearity (A_p_-type system) is evident for the solubility phase profile for the 333 °K samples, suggesting the formation of a higher-order complex with respect to HP-β-CD (*i.e.*, one CCB binds to more than one HP-β-CD). Based on the above, a theoretical calculation was conducted with the assumption that one CCB may form a complex with *n* HP-β-CD molecules; the complexation equilibrium is (Equation (1)):

*D* + *n CD* ↔ *D CD_n_* (1)

where *D* represents the drug CCB and CD is the HP-β-CD molecule. The equilibrium constant K (complexation constant) is expressed as Equation (2):

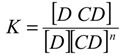
(2)
where [*D*] is the concentration of free drug in the solution, and at saturation, [*D*] is the solubility of the free drug *S*_0_. Equation (2) can be converted to Equation (3):

log(*S_tot_* − *S*_0_) = *n*log[*CD*] + log(*KS*_0_) (3)

Equation (3) indicates that the plot of log(*S*_tot_ − *S*_0_) *versus* log[*CD*] should be a linear curve with a slope close to *n*. Estimation via this approach yielded an *n* of approximately 1.10 (*R*^2^ = 0.991) for the system at 298 °K. This value was close to unity, suggesting that the CCB/HP-β-CD complex in 298 °K samples was largely with the 1:1 stoichiometry. On the other hand, the same estimation gave an *n* of approximately 1.65 (*R*^2^ = 0.976) for the system in 333 °K samples, indicating that higher-order complex(es) formed at 333 °K.

The stability constants of the inclusion complexes were estimated based on the types of the phase solubility profiles. For the A_p_-type system observed for 333 °K samples, assuming that CCB/HP-β-CD complexes of both 1:1 and 1:2 stoichiometry co-existed for 333 °K samples, the stability constants for these two complexes can be estimated via the following expression Equation (4) [10]:

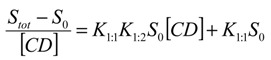
(4)
where *K*_1:1_ and K_1:2_ are the stability constants of the 1:1 and 1:2 inclusion complexes. Equation (4) indicates that the plot of 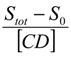 *versus* [*CD*] should yield a linear curve with an intercept of K_1:1_*S*_0_ and a slope of K_1:1_K_1:2_*S*_0_. Through this approach, the K_1:1_ and K_1:2_ for 333 °K samples were estimated to be approximately 6.5 and 12,370.0, respectively, indicating that the 1:2 complex was much more favored for samples that were heated to 333 °K.

For the A_L_-type of profile observed for 298 °K samples, the stability constant of the 1:1 inclusion complex, K_1:1_, can be estimated through the slope of the linear phase solubility profile as the following Equation (5) [5]:

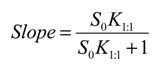
(5)


The K_1:1_ estimated was approximately 4104.5. For the purpose of comparison, we also fitted the solubility phase diagram for 298 °K samples to Equation (4) (*i.e.*, A_p_-type system), which yielded the K_1:1_ and K_1:2_ of approximately 2739.7 and 1.26, respectively. The results show that, in contrast to the 333 °K samples, the 1:1 inclusion complex is clearly favored for 298 °K samples. This suggests that the CCB/HP-β-CD complexation mode changed upon temperature elevation. The K_1:1_ value for the 298 °K samples and the K_1:1_ and K_1:2_ values for 333 °K samples by the method published by Higuchi and Kristiansen [24] and were found to have good agreement with the values estimated by the method published by Loftsson *et al.* [5]. The K_1:1_ for 298 °K samples was estimated to be 4095. For the 333 °K samples, the K_1:1_ and K_1:2_ were 97.8 and 9699.8, respectively.

### 3.2. MD Simulations

**Figure 3:**
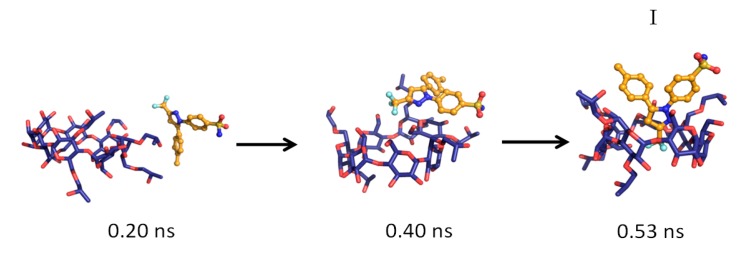
The key steps in the formation of the 1:1 CCB/HP-β-CD inclusion complex (Structure I).

**Figure 4:**
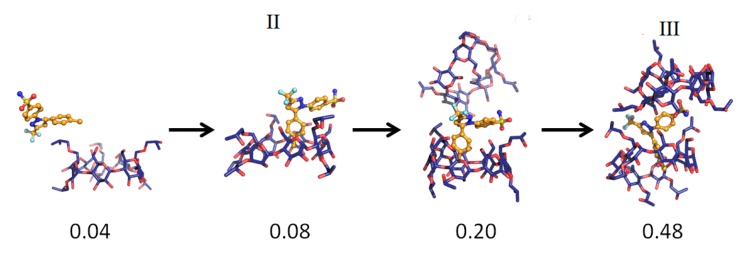
The key steps in the formation of the 1:1 (Structure II) and 1:2 (Structure III) CCB/HP-β-CD inclusion complex.

**Figure 5:**
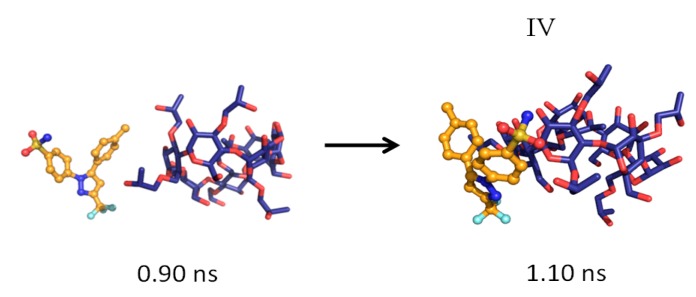
The formation of the 1:1 CCB/HP-β-CD association complex (Structure IV).

**Figure 6:**
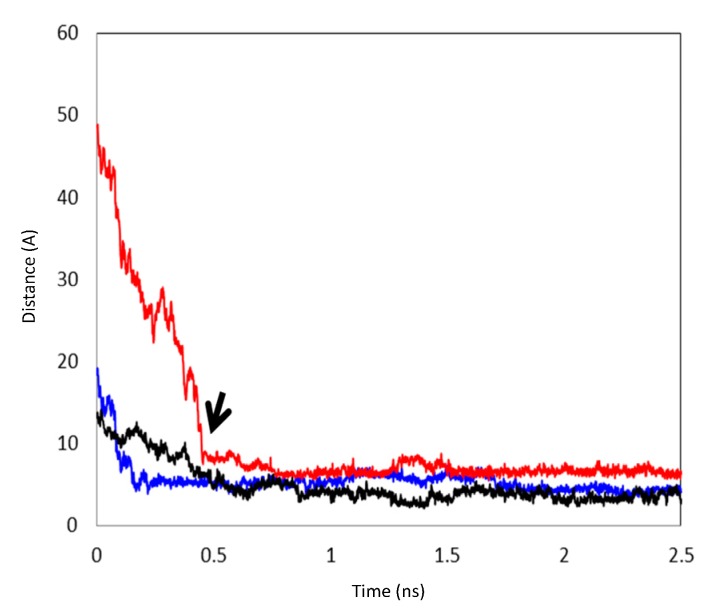
The distances between the centroids of CCB and HP-β-CD during the formation of inclusion complexes. Black: the formation of Complex I; Blue: the distance between the centroids of CCB and the first HP-β-CD during the formation of Complex II and, subsequently, the formation of Complex III; Red: the distance between the centroids of CCB and the second HP-β-CD during the formation of Complex III. The arrow points to the formation of Complex III via Complex II.

The MD simulations and their results are summarized in Table 1. Several CCB/HP-β-CD complex structures were observed, including two different 1:1 inclusion complexes (Structures I and II), one 1:2 inclusion complex (Structure III) and one 1:1 association complex (*i.e.*, CCB was associated with, but not enclosed in, HP-β-CD). The detailed structures are depicted in Figure 3, Figure 4 and Figure 5. A close inspection of the trajectories yielded several observations: (1) Structure I of the 1:1 inclusion complex was formed by the inclusion of the F ring (see Figure 1 for the ring designation) of CCB in the center of HP-β-CD (Figure 3), while Structure II of the 1:1 inclusion complex was formed by inserting the M ring of CCB in the center of HP-β-CD (Figure 4); (2) Structure I was observed relatively frequently (twice at 298 °K and once at 333 °K) and appeared quite stable. Specifically, Structure I was not found to further form a 1:2 CCB/HP-β-CD complex in any of these three MD runs. From this result, it may be speculated that Structure I was unlikely to serve as the intermediate in the formation of the 1:2 CCB/HP-β-CD inclusion complex; (3) on the other hand, Structure II of the 1:1 inclusion complex was observed only at 333 °K and engaged further in the formation of a 1:2 CCB/HP-β-CD inclusion complex (Structure III), indicating that Structure II can be the intermediate for the formation of the 1:2 inclusion complex. Furthermore, it can be seen from Table 1 that only 1:1 CCB/HP-β-CD complexes were observed at 298 °K, while both 1:2 and 1:1 complexes were observed at 333 °K. Despite that the number of simulations runs (*i.e.*, three at each temperature) was too small to draw a statistically representative conclusion on the frequency of occurrence of these complexes, this result in essence conformed to the phase solubility study results wherein an A_L_-type of solubility profile was found at 298 °K, while an A_p_-type of profile was observed at 333 °K. Further discussions on the frequency of occurrence of the complexes will be presented in the next section; (4) It was found that the 1:2 CCB/HP-β-CD inclusion complex (Structure III) observed had the M ring of CCB still embedded in one HP-β-CD, while both the F and S rings were “covered”, but not enclosed in the second HP-β-CD (Figure 4). This can also be seen in Figure 6, where the distances between CCB and HP-β-CD were depicted along the trajectories of the formation of Complexes II and III. Essentially, the distance between CCB and HP-β-CD in Complex II was estimated as 5.297 ± 0.373 Å (averaged during 0.20–0.48 ns of the trajectory). When the second HP-β-CD joined Complex II to form Complex III at around 0.48 ns, the distance between CCB and the first HP-β-CD amounted to a value of 5.120 ± 0.883 Å (averaged from the last 1.5 ns of the trajectory), indicating that the inclusion state of CCB in the first HP-β-CD remained stable. Meanwhile, the distance between CCB and the second HP-β-CD in Complex III gave a value of 6.735 ± 0.538 Å (averaged from the last 1.5 ns of the trajectory), a distance significantly greater than that between CCB and the first HP-β-CD. This supports that the CCB has not been included in the second HP-β-CD. Furthermore, it was also seen that the distance between CCB and HP-β-CD of the 1:1 inclusion Complex I was the shortest, with a value of 3.611 ± 0.604 Å. This is because Complex I was formed by enclosing the F ring of CCB in HP-β-CD, which made the centroid of CCB closer to the center of HP-β-CD; Finally, (5) Structure IV, the 1:1 CCB/HP-β-CD complex with CCB associated with, but not enclosed in, HP-β-CD appeared once at 298 °K and once at 333 °K. This structure is believed to be less stable than the inclusion complex. Nevertheless, some authors did suggest that this could be a solubilization mechanism for CD [5]; hence, this structure was included in the steered MD simulation. Overall, the MD simulation results are consistent with the phase solubility data. The simulation found two different 1:1 CCB/HP-β-CD inclusion complexes (I and II), one of which further engaged in the formation of a 1:2 inclusion complex (III).

**Table 1:** Molecular dynamics simulations for the CCB/HP-β-CD solutions.

Temperature	System ^a^	Simulation Time (ns)	Complexation Structure Observed ^b^	Time When the Final Structure Formed (ns)
298 °K	1	3.0	I	0.53
	2	4.8	IV	1.10
	3	9.0	I	1.30
333 °K	1	8.0	III (via II)	0.48
	2	5.0	I	1.50
	3	9.0	IV	1.60

^a^ The numbering denotes the three starting structures of the CCB/HP-β-CD solution entering the MD simulation (see Section 2.2); ^b^ Structure I: the 1:1 inclusion complex with the F ring of CCB enclosed in HP-β-CD; Structure II: the 1:1 inclusion complex with the M ring of CCB enclosed in HP-β-CD; Structure III: the 1:2 inclusion CCB/HP-β-CD complex; and Structure IV: the 1:1 association complex with CCB associated with, but not enclosed, in HP-β-CD.

### 3.3. Steered MD Simulations

**Figure 7:**
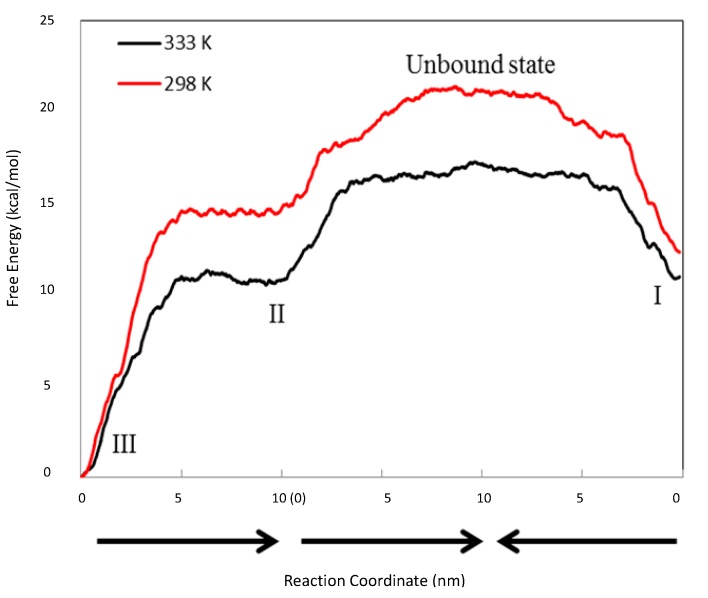
Free energy profile between Complexes I, II, III and the unbound state. The black line is at 298 °K, while the red line is at 333 °K. The reaction coordinate (the *X*-axis) is the distances between the two objects pulled apart in the steered MD runs. The arrows indicate the directions of three steered MD runs, which are from III to II, from II to the unbound state and from I to the unbound state.

The findings from the MD simulations were intriguing. To further understand the conditions under which these complexes were formed, the energetic relationships between these complexes were mapped out via steered MD simulations. Figure 7 depicts the free energy profile between Complexes I, II, III and the unbound state (where CCB and HP-β-CD were detached in the solution). The free energy differences between each complex and the unbound state are listed in Table 2. It is evident from Figure 7 and Table 2 that the unbounded state presented the highest free energy state at both 298 and 333 °K, and hence, the formation of inclusion complexes was energetically favored at both temperatures. This was not surprising and was consistent with the experimental observation. The free energy level of all states found in the MD simulations were ranked as unbound > Complex IV > Complex I > Complex II > Complex III at 333 K. Note that Complex IV, the complex with CCB associated with, but not enclosed in, HP-β-CD, presented at 333 °K a free energy of approximately 3.701 kcal/mol higher than Complex I, which is the complexation state with the next high free energy. The probability ratio between the formation of Complexes I and IV can be estimated through the thermodynamic Equation (6):

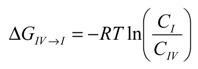
(6)
where Δ*G*_IV→I_ is the free energy difference between I and IV, *R* is the gas constant and 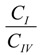 is the ratio between the equilibrium concentrations of I and IV, *C*_I_ and *C*_IV_, respectively, which can also be considered as the probability ratio between the formation of these two complexes, 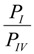, given that the system started from the unbound state. Using Equation (6), the probability ratio between the formation of Complexes I and IV at 333 °K was estimated to be 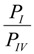 ≈ 269. In other words, the association complex (Complex IV) was less stable relative to Complex I and other inclusion complexation states (which presented still lower free energies). Therefore, it should not be a significant form of complexation in the system. The appearance of the association complex in the MD simulations was likely due to insufficient equilibration time of the MD runs. Because of the finding at 333 °K, we did not perform steered MD simulations on Complex IV at 298 °K assuming a similar instability of Complex IV at 298 °K.

**Table 2:** Free energy differences between the unbound and complexation states.

kcal/mol		*∆G*_Unbound*→*I_	*∆G*_Unbound*→*II_	*∆G*_Unbound*→*III_	*∆G*_Unbound*→*IV_	*∆G*_II*→*I_
298 °K	Average	−8.827	−6.653	−21.166	–	−2.174
	SD	0.062	0.115	0.124	–	0.054
333 °K	Average	−6.249	−6.473	−17.164	−2.548	0.223
	SD	0.051	0.051	0.074	0.038	0.074

It was also found that Complex III (1:2 CCB/HP-β-CD complex) presented the lowest free energy at both temperatures. This finding suggests that the 1:2 complex was the thermodynamically most stable state at both temperatures. The above result appeared inconsistent with the phase solubility data at 298 °K, where no evidence of a significant amount of the 1:2 inclusion complex was observed. This was resolved by a closer inspection of the relative free energy levels of Complexes I and II (Figure 7). The inspection revealed an inversion of energetic level between 298 and 333 °K. Essentially, Complex I presented a lower free energy than II (Δ*G*_II→I_ ≈ −2.045 ± 0.054 kcal/mol) at 298 °K, but a slightly higher free energy than II (Δ*G*_II→I_ ≈ 0.223 ± 0.074 kcal/mol) at 333 °K, indicating that Complex I was more stable than II at 298 °K, but slightly less stable than II at 333 °K. Using Equation (6) to estimate the probability ratio of the formation between Complexes I and II yielded 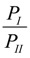 ≈ 39.4 at 298 K and 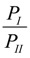 ≈ 0.71 at 333 °K, indicating that at 298 °K, the unbound state predominantly turned into Complex I initially, while at 333 °K, the chances to form Complexes I and II were almost even. Note that for a system starting from the unbound state, the first step is to form 1:1 complexes, *i.e.*, Complexes I and II in this case, while the formation of a 1:2 complex needs to go through a 1:1 complex. Therefore, the probability ratio between Complexes I and II determines which 1:1 complex is likely to appear initially from the unbound state, irrespective of whether it is a metastable state.

The finding above is interesting in that Complex I, unlike Complex II, was not found to further evolve to a 1:2 CCB/HP-β-CD complex in the MD simulations, despite that it appeared in three runs with a total running time of 17 ns (Table 1). We thus hypothesized that Complex I may be unable to directly evolve to a 1:2 complex unless it converts to Complex II, a transition that has to overcome the high-energy barrier presented by the unbound state. Given this hypothesis, if the energy barrier between Complex I and the unbound state is sufficiently large, the system at 298 °K may be trapped in the metastable state of Complex I once formed. This could explain that the phase solubility curve of CCB for 298 °K samples was of the A_L_-type (first-order complexation with HP-β-CD). Furthermore, the phase solubility curve was sensitive to temperature elevation, providing further evidence to support that the “observed state” of the system at 298 °K could be a metastable one. The lifespan of this metastable state is determined by the magnitude of the energy barrier (the activation energy *E*_a_) and temperature *T* following the Arrhenius equation 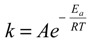, where *k* is the reaction rate, *A* is the frequency factor and R is the gas constant. Assuming *A* is temperature independent, we may use the Arrhenius equation to estimate the reaction rate change upon temperature elevation (Equation (7)):

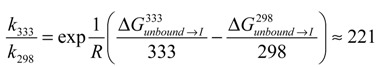
(7)


That is, the conversion rate for Complex I to II was increased approximately 221 times upon a temperature increase from 298 to 333 °K and further explains the temperature activation of the formation of the stable 1:2 CCB/HP-β-CD complex.

## 4. Conclusions

Hydroxypropyl-β-cyclodextrin (HP-β-CD) is commonly used as complexation reagent to solubilize compound for *in vivo* dosing. However, the degree of solubility enhancement for dug candidates was often limited by the low K constant and only forming the 1:1 complex. In many cases, it is known that the efficiency of the solubilization of drugs in HP-β-CD can be significantly improved by the formation of a 1:2 complex and become much more useful to prepare formulations. However, despite the solubility advantage of the formation of the 1:2 complex, the overall understanding of the inclusion mechanism of forming the 1:2 complex is ambiguous. Additionally, the ambiguity arising from the determination of the complexation stoichiometry [5] hinders researchers from better utilizing such a system. Therefore, it is crucial to clarify and reconcile these seemingly contradictory observations so that a unifying understanding of the complexation mechanism can be delivered. We believe more understanding of this behavior is critical for researchers in drug delivery. In this study, we pioneer the usages of molecular dynamics (MD) simulation to probe the structural and energetic features of the complexation process of a model drug of celecoxib (CCB) and HP-β-CD. The simulation results suggest that a transition state of CCB and HP-β-CD may exit at a higher temperature of CCB and HP-β-CD; a model drug, such as celecoxib (CCB), that is known to form 1:1 complexes, can achieve a higher degree of complexation (1:2) and obtain much improved solubility when the same amount of cyclodextrin was used. We believe this finding could serve as a model system that can provide important insights into the inclusion mechanism. The study finds evidence strongly in favor of the MD simulation help in understanding the complexation of the CCB and HP-β-CD system, and a similar approach should be investigated for future usages of MD.
